# Paralysie faciale bilatérale : une présentation atypique d’une maladie de Lyme (cas clinique)

**DOI:** 10.11604/pamj.2024.47.156.43116

**Published:** 2024-04-03

**Authors:** Sophie Collignon, François Coenen, Pierre Mols, Caroline Chauvin, William Ngatchou

**Affiliations:** 1 Service des Urgences, Réseau Iris Sud, Université Libre de Bruxelles, Bruxelles, Belgique,; 2 Service des Urgences et de Chirurgie Cardiaque, Hôpital St Pierre de Bruxelles, Université Libre de Bruxelles, Bruxelles, Belgique

**Keywords:** Paralysie faciale, maladie de Lyme, neuroborréliose, monoplégie faciale, cas clinique, Facial palsy, Lyme disease, neuroborreliosis, facial monoplegia, case report

## Abstract

La neuroborréliose de Lyme est une zoonose rare dont le diagnostic peut se révéler difficile particulièrement en faible zone endémique. Nous rapportons le cas d´un homme de 35 ans avec un tableau de dorsalgies invalidantes précédée d´une monoplégie faciale traitée à tort comme une paralysie a frigore puis comme une lombosciatalgie post traumatique. L´apparition d´une diplégie faciale permettra enfin d´évoquer et de confirmer le diagnostic. L´administration de ceftriaxone permettra la résolution progressive des symptômes.

## Abstract (en)

Lyme neuroborreliosis is a rare zoonosis which can be difficult to diagnose, in particular in low endemic areas. We here report the case of a 35-year-old man presenting with disabling back pain preceded by facial monoplegia, which was wrongly treated as Bell’s palsy (paralysis a frigore) and then as post-traumatic lumbosciatica. The onset of facial diplegia allowed for a definitive diagnosis. The patient was treated with ceftriaxone and symptoms gradually improved.

## Introduction

La neuroborréliose de Lyme est une zoonose transmise à l´homme par la piqûre de la tique Ixodes ricinus infectée par le spirochète Borrelia burgoferi stricto [1]. Les tiques vivent essentiellement dans les forêts les prairies mais aussi dans les zones boisées et des parcs urbains et périurbains [1]. L´incidence de cette pathologie est variable dans le monde et même dans un pays, elle varie d´une région à l´autre. Au Canada par exemple, le Québec, l´Ontario et la Nouvelle Ecosse; aux USA, les Etats du Nord-Est et du nord du MidWest étaient les plus touchées [1,2]. En Belgique, selon la Biodiversité Walonniebe, environ 11000 personnes consultent par an pour un érythème migrant et entre 200 et 300 personnes hospitalisées pour maladie de Lyme [3].

Le risque est accru en mai et octobre quand les températures oscillent entre 10 et 12 degré; favorables à l´activité des tiques [3]. Cette pathologie évolue classiquement en trois phases. La phase précoce localisée pouvant survenir 3 à 30 jours après la piqûre et se caractérisant par un érythème migrant pouvant être accompagné d´un état grippal, de polyadinopathie et d´arthralgies diffuses [1,4,5]. Dans 90% de cas ses manifestions disparaissent spontanément de manière favorable en 3 à 4 semaines voir quelques mois [1,4]. La phase secondaire qui concerne environ 15% de patients apparait quelques semaines à quelques mois après la contamination et est caractérisée par des manifestations multisystémiques notamment articulaires (mono ou oligo-ararthrite touchant les grosses articulations); neurologiques (méningo-radiculite, paralysie faciale, syndrome méningé); cardiaques (myopéricardite) [4-6].

La phase tardive débute de 6 mois à quelques années après le début de l´infection traitée ou pas et se caractérise par le passage à la chronicité des lésions neurologiques (encéphalomyélite, polyneuropathie); cutanées (acrodermatite chronique atrophiante); articulaires (arthrite récidivante chronique) [4-6]. Nous rapportons le cas d´un patient de 35 ans avec une présentation clinique pernicieuse ayant différé le diagnostic de neuroborréliose de Lyme.

## Patient et observation

**Information du patient:** un homme de 35 ans se présente la nuit aux urgences pour des dorso-lombalgies aiguës connues depuis 8 semaines mais en aggravation et ce malgré un traitement antalgique optimal. Il a relaté un banal traumatisme au surf 5 semaines avant l´apparition des douleurs. Il a déjà consulté par deux fois pour ses plaintes, une radiographie de la rachi dorso-lombaire avait été faite et n’avait montré aucune anomalie. Des séances de kinésithérapies avaient été prescrites sans amélioration.

Il décrit aussi l´apparition d´un bouton de fièvre quelques jours avant l´apparition des dorsalgies. Le patient n´a aucun ATCD particuliers et a une consommation modérée de tabac, d´alcool et de cannabis. L´intensité des douleurs est plus importante la nuit et le patient dort peu. Ces douleurs sont décrites comme des brûlures intenses.

**Résultats cliniques:** l´examen clinique aux urgences révèle une disparition de toute mimique du visage. Le patient dit avoir consulté son médecin généraliste pour ce problème une semaine auparavant; celui-ci avait conclu à une paralysie a frigore due à un virus et aucun bilan diagnostic et traitement ciblé n´avait été initié. La paralysie du visage était au départ unilatéral à gauche au moment de la consultation mais était devenue bilatérale 48 heures après. Le patient ne s´était inquiété outre mesure car médecin traitant l´avait rassuré le patient sur le caractère banal de l´affection d´autant plus que les douleurs lombaires nocturnes le gênaient plus.

**Démarche diagnostique:** l´anamnèse décrit une ouverture difficile des yeux sans trouble visuel, une modification du goût et une absence de céphalée. L´examen clinique montre un patient apyrétique avec une diplégie faciale avec un sigle de Bell présent bilatéralement. Il n´y a pas déficit sensitif au niveau du visage. Les mouvements oculaires sont normaux sans nystagmus. Les pupilles sont isocores et réflectiques.

Il présente une légère raideur de nuque sans déficit de force au niveau des 4 membres. Ses réflexes sont modérément diminués aux 4 membres (2/4). Le reflexe cutanéo-plantaire est en flexion bilatérale. Le Barré tenu et sa marche est normale. Il a une hyperesthésie au niveau dorsolombaire avec Lasègue présent à 60° bilatéralement.

**Intervention thérapeutique:** au vu des éléments décrits ci-dessus, tomodensitométrie lombaire avant ponction lombaire est proposée et s´avère normale.

La ponction lombaire est limpide et démontre la présence de 29 éléments nucléés à prédominance lymphocytaire (75%) au niveau du liquide céphalo-rachidien (LCR) et la présence très élevée d´immunoglobulines type Immunoglobulin M (IGM) de Borrélia au niveau du LCR (> 190 UA/ml) ainsi qu´une protéinorachie à 3, 662 g/L.

La PCR Herpès simplex 1 et 2 est négative. La sérologie *Herpes simplex virus* (HSV) et *Herpes zoster virus* (HZV) sont négatives. La sérologie Borrelia est douteuse pour l´IGM est douteuse et positive pour l´Immunoglobulin G (IGG).

**Suivi et résultats:** nous avons conclu à une neuroborréliose en phase disséminée précoce; avec diplégie faciale, une méningite lymphocytaire et dorso-lombaire comme manifestations.

La rétro-anamnèse policière révèle que le patient a séjourné 3 mois avant dans les Landes et avoir été piqué par des moustiques et possiblement par une tique. Il décrit aussi avoir présenté un érythème au niveau de l´aisselle qui aurait duré 4 semaines mais étiqueté de frottement lié à la sueur.

Le patient a dès lors bénéficié initialement et rapidement d´une antibiothérapie pendant 14 jours à base de Ceftriaxone 2 grammes intra-veineuse par jour associé d´Aciclovir au début; ce dernier étant interrompu dès l´obtention de la négativité des sérologies herpès. Après quelques jours, on remarque une amélioration des symptômes tant au niveau de la paralysie que des douleurs. Après quatre semaines, l´examen neurologique s´est normalisé avec une récupération totale de la diplégie.

**Consentement éclairé:** le patient a donné son consentement éclairé.

## Discussion

Notre cas révèle le challenge diagnostique que représente cette pathologie. Bien que compréhensible en zone endémique faible comme à Bruxelles, Gasmi *et al*. ont décrit 65,5% d´érythèmes migrants loupés par les généralistes au Québec; zone pourtant à prévalence importante [1]. Le généraliste ayant examiné notre patient au début avec une paralysie faciale unilatérale avait conclu à tort en une paralysie a frigore sans tout autre bilan. Dans cette entité clinique, la paralysie en moins de trois jours et ne sont pas accompagné de signe général. Il est donc clair qu´une anamnèse approfondie relevant la présence d´un bouton de fièvre et une balade en forêt des Landes auraient pu suggérer le diagnostic. Une atteinte faciale de type centrale, une atteinte faciale bilatérale, l´apparition lente (>2 semaines); les comportements sexuels à risque (VIH, syphilis), la baisse de l´audition , les vésicules douloureuses du conduit auditif externe, les signes méningés, une maladie systémique, un traumatisme, une variabilité dans le temps sont d´autres éléments qui associés à une paralysie faciale devraient faire douter du diagnostic de paralysie a frigore [7]. Les diagnostics différentiels à évoquer en cas de paralysie faciale sont la neuroborréliose de Lyme, l´atteinte centrale, la neuro sarcoïdose, la myasthénie grave, la neuro-syphilis ou HIV, les atteintes périphériques bilatérales du Guillain Barré ou Miller Fisher, le syndrome de Melkersson-Rosenthal, les infections du système nerveux central, le zona otique, une lésion post traumatique du nerf facial [7].

Bien l´accident léger au surf ait pu participer à la mauvaise appréciation de la dorso-lombalgie; symptomatologie en avant plan du tableau clinique, les caractéristiques de cette douleur ainsi la chronologie des évènements auraient due alerter. En effet l´intensité de la douleur pas en rapport avec la légèreté de l´accident, sa typologie «brûlure», la raideur légère de la nuque et la diminution des réflexes ostéo-tendineux étaient des signes cliniques incohérents avec le diagnostic de simple contusion lombaire.

Au final c´est la paralysie faciale bilatéral qui aura été l´élément déterminant ayant permis de retracer la chronologie des événements et d´orienter vers le diagnostic. La présence d´une paralysie faciale bilatérale 2 mois après la piqûre supposée associée à une symptomatologie articulaire classe notre patient dans la phase secondaire. Bien que le diagnostic soit avant clinique, la sérologie peut aider à confirmer le diagnostic. Le test sérologique sanguin a une sensibilité entre 70 et 100%. Elle recherche les IgM et les IgG. Ces dernières apparaissent au-delà de 6 semaines contre 2 et 4 semaines les IgM. On pourrait donc avoir une sérologie négative en phase précoce même avec un érythème migrant. Compte tenu du risque de faux positifs, en pratique, en cas de sérologie positive, un test de confirmation par western blot est conseillée. L´analyse du liquide céphalorachidien peut aider dans la confirmation diagnostic en cas de symptomatologie neurologique car en plus d´exclure les autres causes infectieuses, on peut y détecter les IgG et faire la *polymerase chain reaction* (PCR). Toutes les manifestations cliniques de la borréliose doivent faire l´objet d´une antibiothérapie ciblée [8]. L´objectif étant de limiter l´aggravation des symptômes et la progression vers les stades tardifs [8,9].

Par contre une sérologie positive sans symptomatologie spécifique est une séquelle sérologique qui ne doit pas être traitée. Le traitement des formes localisées repose sur la doxycycline 100 mg deux fois par jour en premier choix pendant 10 jours (4 mg/kg/jour en 2 prises chez l´enfant; max 10mg par prise); amoxicilline 500mg 3x/j en deuxième choix et ce pendant 14 jours (50 mg/kg/jour en 3 prises chez l´enfant; max 500 mg par prise) [4,8,9]. Quant aux formes disséminées précoces, la Doxycycline 100 mg 2x/jour ou la Ceftriaxone 2g/j pendant 14 jours sont les traitements recommandés [8,9]. Dans les formes disséminées tardives, la Doxycycline et la Ceftriaxone seront prolongées pendant 28 jours avec la Ceftriaxone comme premier choix en cas de neuroborréliose [8,9].

## Conclusion

La maladie de Lyme est une zoonose dont la présentation clinique aux urgences est multiforme. Seuls une anamnèse fouillée et une examen clinique détaillé pourraient limiter l'errance diagnostic particulièrement dans les atteintes neurologiques de cette maladie.
